# Axonal and myelinic pathology in 5xFAD Alzheimer’s mouse spinal cord

**DOI:** 10.1371/journal.pone.0188218

**Published:** 2017-11-27

**Authors:** Tak-Ho Chu, Karen Cummins, Joseph S. Sparling, Shigeki Tsutsui, Craig Brideau, K. Peter R. Nilsson, Jeffrey T. Joseph, Peter K. Stys

**Affiliations:** 1 Hotchkiss Brain Institute, Department of Clinical Neurosciences, University of Calgary, Calgary, Alberta, Canada; 2 Department of Physics, Chemistry and Biology, Linköping University, Linköping, Sweden; 3 Departments of Clinical Neurosciences, Alberta Health Services, Calgary, Alberta, Canada; 4 Pathology and Laboratory Medicine, Alberta Health Services, Calgary, Alberta, Canada; Torrey Pines Institute for Molecular Studies, UNITED STATES

## Abstract

As an extension of the brain, the spinal cord has unique properties which could allow us to gain a better understanding of CNS pathology. The brain and cord share the same cellular components, yet the latter is simpler in cytoarchitecture and connectivity. In Alzheimer’s research, virtually all focus is on brain pathology, however it has been shown that transgenic Alzheimer’s mouse models accumulate beta amyloid plaques in spinal cord, suggesting that the cord possesses the same molecular machinery and conditions for plaque formation. Here we report a spatial-temporal map of plaque load in 5xFAD mouse spinal cord. We found that plaques started to appear at 11 weeks, then exhibited a time dependent increase and differential distribution along the cord. More plaques were found in cervical than other spinal levels at all time points examined. Despite heavy plaque load at 6 months, the number of cervical motor neurons in 5xFAD mice is comparable to wild type littermates. On detailed microscopic examination, fine beta amyloid-containing and beta sheet-rich thread-like structures were found in the peri-axonal space of many axons. Importantly, these novel structures appear before any plaque deposits are visible in young mice spinal cord and they co-localize with axonal swellings at later stages, suggesting that these thread-like structures might represent the initial stages of plaque formation, and could play a role in axonal damage. Additionally, we were able to demonstrate increasing myelinopathy in aged 5xFAD mouse spinal cord using the lipid probe Nile Red with high resolution. Collectively, we found significant amyloid pathology in grey and white matter of the 5xFAD mouse spinal cord which indicates that this structure maybe a useful platform to study mechanisms of Alzheimer’s pathology and disease progression.

## Introduction

Alzheimer’s disease (AD) is a neurodegenerative disease characterized by the accumulation of amyloid plaques and neurofibrillary tangles in various brain regions, depending on disease stage [1]. Most studies have traditionally focused on the brain, with far less attention paid to other areas of the central nervous system (CNS), such as the spinal cord. However, it has been shown in AD patients that beta amyloid plaques [2,3], neurofibrillary tangles and hyperphosphorylated tau [4,5] can accumulate in the spinal cord. What is not known is how much AD pathology of the spinal cord contributes to the functional disturbances such as gait, balance, bowel and bladder dysfunction commonly seen in late stage AD patients. The critical role of the spinal cord within the CNS, and its seeming propensity to accumulate AD pathology, warrants further investigation. Moreover, its long anatomy, large-diameter axons, well organized white matter tracts, and segmental nature may help to gain a better mechanistic view of the development and progression of the disease such as amyloid protein transfer between neurons, axonopathy [6] and white matter pathologies.

Animal model systems have proven to be very useful in the exploration of AD pathologies and therapeutic strategies [7,8]. In our study, we used 5xFAD mice which develop extensive and rapid amyloid pathology in the brain [9]. The 5xFAD strain carries 5 human familial AD gene mutations, with 3 in the gene encoding amyloid precursor protein (APP) and the other two in the presenilin-1 gene. 5xFAD mouse exhibits many abnormalities including intraneuronal beta amyloid accumulation, activation of astrocytes and microglia, reduction in synaptic protein, neuronal loss in cortical layer 5 and subiculum with caspase-3 activation, and cognitive decline [9,10]. The relatively rapid accumulation of AD pathology makes this mouse line attractive to study pathophysiological mechanisms.

Previous studies using different strains of transgenic mice harbouring human amyloid precursor protein and/ or presenilin mutations mention deposition of plaques in the spinal cord [11–13], with tangles also present if tau mutations co-exists [14]. However, little is known about the systematic accumulation of cord pathology in AD transgenic mice. The aim of our study was to examine the 5xFAD mouse spinal cord in a spatial and temporal manner to gauge its potential for use as a model for the development of AD pathology. Furthermore, the structural advantages of spinal axons may facilitate the detection of subtle pathological amyloid deposition that has not been reported previously. Indeed, we found non-uniform distribution of amyloid plaques along the spinal cord. We also show that spinal axons contain fine thread-like deposits of beta amyloid, together with micro-structural changes in the myelin sheath, suggesting that axonopathy and myelinopathy, driven by subcellular accumulation of beta amyloid, might play a role in the pathophysiology of AD, not only in the spinal cord, but throughout the CNS.

## Materials and methods

### Animals

All animal experiments were approved by the Animal Care Committee at the University of Calgary. All applicable international, national, and institutional guidelines for the care and use of animals were followed. 5xFAD mice (Tg6799, stock number 34840-JAX) were purchased from the Jackson Laboratory (Bar Harbor, ME, USA), the colony was maintained by crossing heterozygous transgenics with wild type mice. Genotyping was performed by polymerase chain reaction analysis of ear sample DNA according to Jackson Laboratory’s instructions. Wild type littermates were used as controls. A total of 34 mice were used in the study.

### Perfusion and tissue processing

Animals were given a lethal dose of sodium pentobarbital and perfused intracardially with normal saline followed by ~50ml of fixative containing 4% paraformaldehyde in 0.1M phosphate buffer (pH 7.4). The brain and spinal cord were harvested, post-fixed with fresh fixative overnight, and subsequently placed in 30% phosphate buffered sucrose. After the samples had sunk, the cords were embedded in optimum cutting temperature compound (VWR, Suwanee, GA, USA), frozen in pre-cooled isopentane, and cut in cross or sagittal sections at a thickness of 10 μm on a cryostat. The sections were collected on SuperFrosted Plus slides (VWR, Chester, PA, USA) and stored at -20°C for later use.

### Immunohistochemistry

We used anti-beta amyloid (1–16) clone 6E10 and anti-beta amyloid (1–42, AB42) antibodies to label beta amyloid species. Antigen retrieval with formic acid was performed unless otherwise noted. Briefly, sections were washed with 0.01M phosphate buffered saline (PBS, pH 7.4) and treated with 98% formic acid for 5 minutes at room temperature. After washing with PBS, the sections were incubated in blocking solution containing 10% normal serum, 1% bovine serum albumin and 0.3% triton X-100 for 1 hour at room temperature. The sections were subsequently incubated in primary antibodies at 4°C for 14–16 hours. Primary antibodies (Table 1) were omitted in secondary-only controls. After three washes (15 min each) in PBS-tween 20 (0.05%, PBS-T), the sections were incubated in the corresponding fluorescent conjugated secondary antibodies (1:500, Invitrogen) in PBS for 1 hour at room temperature. After three washes in PBS-T, sections were mounted with Fluoromount mounting medium (Cedarlane, Burlington, ON, Canada).

**Table 1:** Primary antibodies used in the study.

Primary Antibody	Source	Catalog Number	Dilution
Beta amyloid (1–16), clone 6E10	Covance	SIG-39320	1:1000
Beta amyloid (1–42)	Millipore	AB5078P	1:200
Choline acetyltransferase (ChAT)	Millipore	AB144P	1:200
Ionized calcium binding adaptor molecule (IBA-1)	WAKO	019–19741	1:200
Glial fibillary acidic protein (GFAP)	DAKO	Z0334	1:500
Nogo-A	Millipore	AB5664P	1:200
Neurofilament 200kDa (NF)-rabbit	Sigma	N4142	1:1000
Neurofilament 160kDa (NF)-chicken	Novus Biologicals	NB300-222	1:1000
Amyloid precursor protein (APP)	Abcam	Ab2072	1:100
Myelin Basic Protein (SMI99)	Covance	SMI-99P	1:1000
CNPase	Covance	SMI-91R	1:500
Tyrosine Hydroxylase (TH)	Millipore	AB152	1:200
Lysosomal-associated membrane protein 1 (LAMP1)	BD Pharmingen	553792	1:200
Phospho-tau (Ser202, Thr205), clone AT8	Invitrogen	MN1020	1:200

For co-labeling of myelin basic protein (SMI 99) and beta amyloid, we achieved better results with a 2-step staining procedure. Briefly, sections were treated with formic acid and labeled with anti-beta amyloid (AB42) and anti-neurofilament (chicken host) antibodies followed by secondary antibodies as described above. Then we delipidated the sections in acetone for 90 seconds followed by three washes in PBS. We then incubated the sections in SMI 99 antibody at 4°C for 14–16 hours followed by fluorescent conjugated goat anti-mouse secondary antibody. After three washes in PBST, sections were mounted with Fluoromount.

All images were acquired with a Nikon A1 confocal microscope equipped with spectral detector and a 25X water dipping objective (NA = 1.1, Nikon, Japan).

### Quantitation of beta amyloid plaques

Cervical (C7-C8), thoracic (T12) and lumbar (L4-5) spinal cross sections from 5xFAD and wild type mice at different ages (n = 3 for 11 week; n = 4 for 19 week; and n = 5 for 27 week old mice) were immunolabeled with 6E10 and fluorescent conjugated antibodies. Five sections were used for each sample. Whole spinal cross-sections were scanned with a slide scanner (Olympus VS120-S5, Tokyo, Japan). They were then imported into Fiji (https://fiji.sc/) and whole spinal cord (grey and white matter) analyzed using the particle analyzer function with thresholding.

### Counting of motor neurons

C7-C8 spinal sections from 27 week-old wild type and 5xFAD mice (n = 5 in each group) were immunolabeled with anti-choline acetyltransferase (ChAT), a motor neuron marker, as described above. Eleven sections each spaced 200μm apart were counted for each sample. All ChAT positive motor neurons in the ventral horn were counted. The total numbers of counted motor neurons in each group was compared using Student’s *t* test.

### TUNEL staining and co-labeling

Apoptotic cells in 5xFAD spinal cross-sections were labeled with the ApopTag fluorescein in situ apoptosis detection kit (Catalog number S7110, Millipore, Bedford, MA, USA) according to manufacturer’s instructions. Briefly, sections were post-fixed using a 2:1 ratio of ethanol and acetic acid at -20°C for 5 minutes followed by 3 washes with PBS. They were then exposed to an enzyme (terminal deoxynucleotidyl transferase) which adds digoxigenin-conjugated nucleotides to the DNA strand breaks at 37°C for 1 hour. The enzymatic reaction was stopped with stop wash buffer. The sections were washed, then incubated in fluorescein-conjugated anti-digoxigenin along with other primary antibodies for 1 hour at room temperature. After three washes with PBS, sections were incubated in appropriate secondary antibodies for 30 minutes at room temperature, then washed 3x with PBS and mounted with 4',6-diamidino-2-phenylindole (DAPI) containing ProLong Gold mounting medium (Molecular Probes, Eugene, OR, USA).

### Fluorescent amyloid probes

We used three conformationally sensitive amyloid probes to confirm the identity of the beta amyloid threads. The staining protocols used were slightly modified from those in the literature. Briefly, for pentamer formyl thiophene acetic acid (pFTAA) staining [15], 3μM of pFTAA dissolved in 0.01M PBS (pH7.4) was applied to tissue on slides for 60 minutes at room temperature. For thioflavin T (ThT, Abcam, Cambridge, MA, USA) staining [16], 0.5% ThT dissolved in 1N hydrochloric acid was applied for 5 minutes at room temperature. The sections were then rinsed three times with PBS using a disposable transfer pipette and mounted with Fluoromount.

For 4,4'-[(2-Bromo-1,4-phenylene)di-(1*E*)-2,1-ethenediyl]bisphenol (K114, Tocris, Ellisville, MO, USA) staining, 150μM K114 [17] was dissolved in 1:1 ratio of dimethyl sulfoxide (DMSO) and 0.1M sodium bicarbonate buffer (pH 10.5) and applied to tissue on slides for 30 minutes at room temperature. The sections were then rinsed three times with sodium bicarbonate buffer and mounted with 1:1 ratio of Fluoromount and sodium bicarbonate buffer to maintain pH at 10.5. Images were taken with a Nikon A1 confocal microscope equipped with a spectral detector for K114 spectral analysis.

For co-labeling of K114 and AB42, K114 staining was performed and spectral images were acquired before immuno-labeling with AB42 since formic acid disrupts the amyloid structure required for K114 binding. The same area was re-imaged and the resulting micrographs were registered and merged for colocalization analysis.

### Spectral processing and analysis

Spectral images were imported into ImageTrak (http://www.ucalgary.ca/styslab/imagetrak, written by P.K.S.) for processing and analysis. The image was divided into equally sized subregions, or “kernels”, consisting of a square of 3x3 pixels. The average spectrum was calculated from all pixels within a kernel to increase the signal-to-noise ratio. To numerically quantify K114 spectral shifts, two ‘extreme’ reference (bracketing) spectra were generated from K114-stained wild type mouse samples (using non-plaque background fluorescence) and plaque-containing 5xFAD mice. For each kernel a non-linear spectral transformation algorithm was used to calculate an index reflecting how closely each kernel’s spectral shape resembles either of the two extreme bracketing spectra. The indexes were then pseudo-coloured into a heat map from violet to red, for a visual representation of the spectral ranges in the image.

### Nile Red staining and quantitaion of myelin abnormalities

Sagittal tissue sections were washed with 0.01M PBS, then Nile Red was applied at 20 μM for 5 minutes at room temperature. The sections were then rinsed in PBS and imaged by spectral confocal. To count the number of myelin abnormalities, animals from 19 week-, 27 week-, 37 week- old 5xFAD mice, and 37-week old WT mice (n = 3 in each group) were used. Fields of 250 μm x 250 μm images with 500 μm apart between images were taken with 25X lens and motorized stage. At least 5 fields were taken from each section and at least 2 sections were used for each sample. Abnormalities include myelin spheres and myelin thickening were counted by a blinded observer. For co-labeling of Nile Red and AB42, Nile Red staining was performed and spectral images were acquired before immuno-labeling with AB42 and counterstaining with DAPI. The same area was re-imaged and micrographs were registered and merged for analysis.

### Statistical analysis

Results are expressed as mean ± standard error of the mean (SEM). One-way analysis of variance and the Tukey multiple comparisons test or Student’s *t*-test, where appropriate, were used to determine the statistical significance of differences among the means. A value of p <0.05 was considered significant.

## Results

### Beta amyloid plaques accumulate in grey and white matter in 5xFAD spinal cord

**Fig 1:**
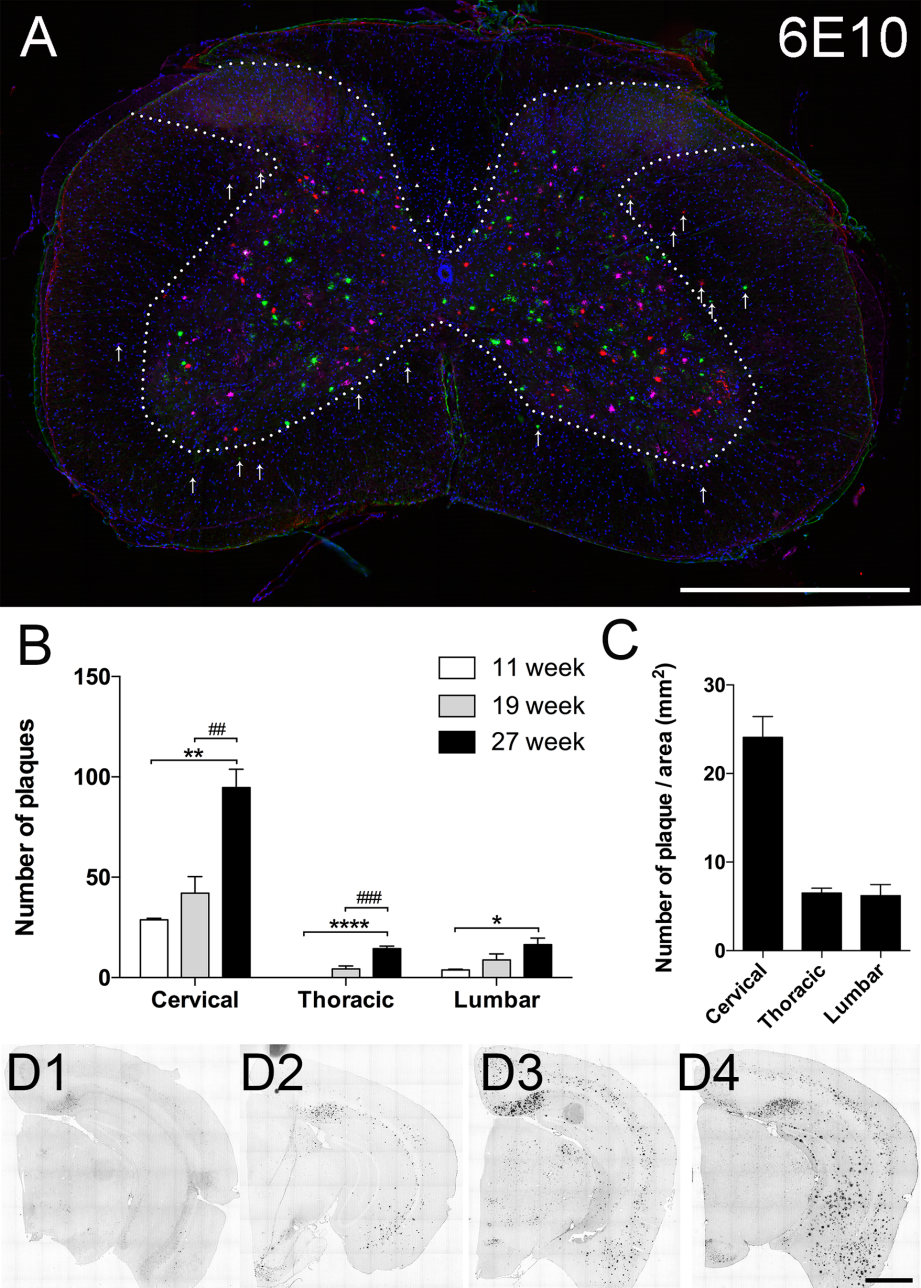
Beta amyloid plaque deposition in the spinal cord. (A) Representative stacked image consisting of five cervical cord cross-sections immuno-labeled with 6E10 antibody. Each section is 100μm apart and is represented by one colour. Majority of the amyloid plaques are deposited in the grey matter except Rexed laminae I and II. Some plaques are located in the white matter, with a small number distributed in the lateral and ventral white matter (arrows); most were found in the ventral part of the dorsal column (arrowheads), which corresponds to corticospinal tract in rodents. (B) Quantitative analysis of number of 6E10-positive beta amyloid plaques at the cervical (C7-C8), thoracic (T12) and lumbar (L4-L5) spinal levels at different time points. Results from 6 and 8 week old mice are omitted since no plaques were found at those time points. There was an age-dependent increase in the number of plaques in whole spinal cord at each level, however cervical spinal cord always had the most plaques regardless of age. (C) To control for the difference in cross-sectional area at different levels of the spinal cord, we also calculated the density of plaques (expressed as number of plaque/ total spinal cord area) at 27 weeks of age. Representative 6E10 labeled brain sections from 8 week (D1), 11 week (D2), 19 week (D3), and 27 week (D4) old 5xFAD mice also show progressive increase in number and size of amyloid plaques. Scale bar in A and D = 1mm.

Cross-sections of spinal cords from 27 week old 5xFAD mice immuno-labeled with 6E10 antibody revealed amyloid plaques primarily deposited in ventral and dorsal grey matter except Rexed lamina layers I and II (Fig 1A). A few plaques were also found in spinal white matter, particularly in the ventral part of the dorsal column that corresponds to the corticospinal tract in rodents.

We then examined the spatial-temporal distribution of beta amyloid plaques. We surveyed the brain and spinal cord at 6, 8, 11, 19 and 27 week old mice. As previously reported [9], 6E10 positive plaques were found in 5xFAD mouse brains in all samples older than 6 weeks (Fig 1D). In contrast, plaques were not observed in the spinal cord of 5xFAD mice until 11 weeks of age, when they were found only in cervical and lumbar regions. At 19 and 27 weeks, plaques were found along the entire length of the spinal cord, primarily in the grey. We quantified the number of plaques at the 7^th^-8^th^ cervical, 12^th^ thoracic and 4^th^-5^th^ lumbar segments at 11, 19, and 27 weeks of age and found that plaque number increased over time at each level. Notably, the cervical spinal cord had by far the highest number of plaque per section (Fig 1B) or plaque density (Fig 1C) at all ages examined.

### Beta amyloid plaques are not toxic to spinal motor neurons

**Fig 2:**
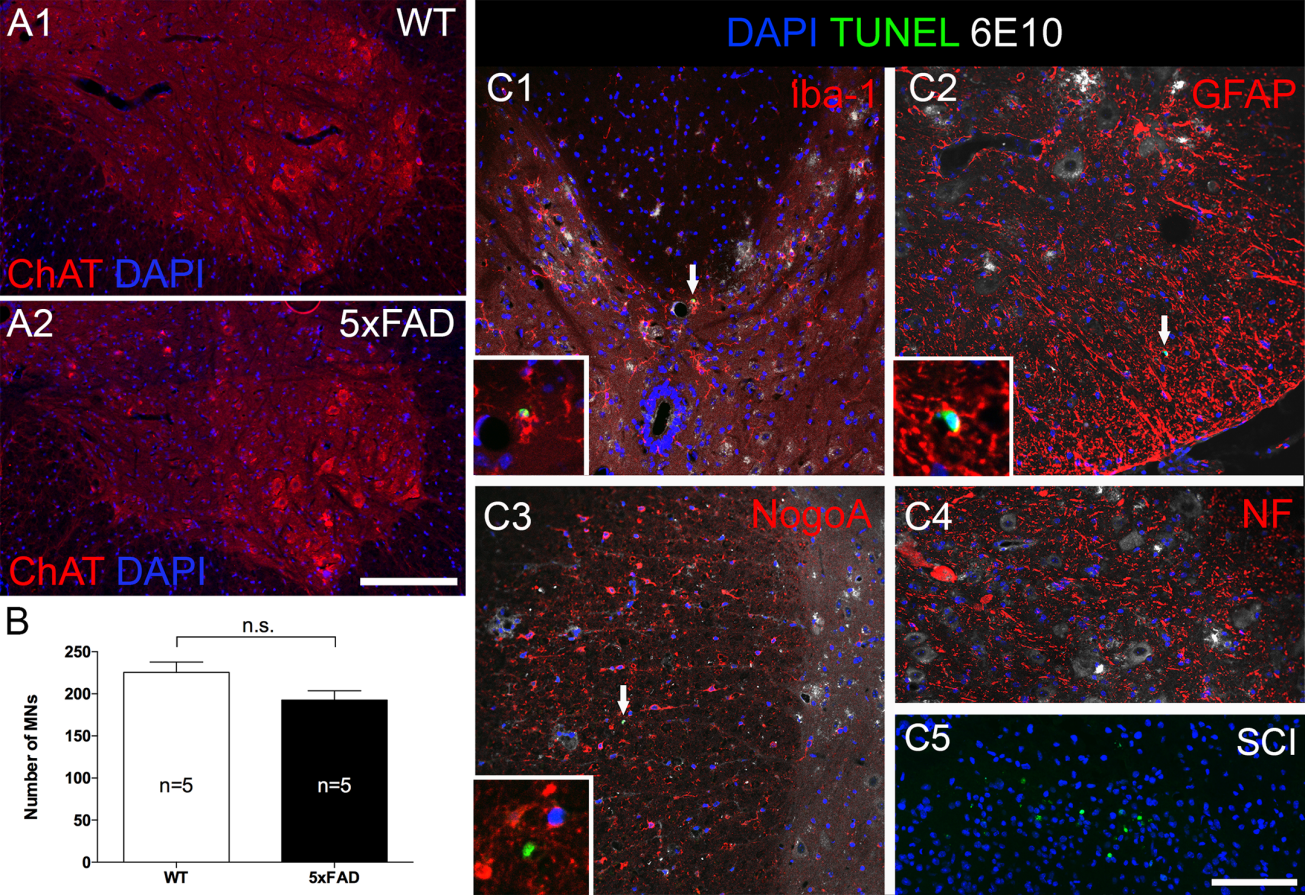
Despite heavy plaque load, beta amyloid plaque did not cause significant motor neuron loss at 27 weeks of age in 5xFAD mice. (A) Representative micrographs showing ventral horns from a 5xFAD mouse (A2) and a wild type littermate (A1). Sections were labeled with anti-choline acetyltransferase (ChAT), a motor neuron marker, and counter-stained with DAPI to label nuclei. Motor neurons in both strains appeared normal with centrally placed nuclei and no sign of atrophy or chromatolysis. (B) Counting of ChAT-positive motor neurons revealed similar numbers in both strains. (C) Results were further confirmed by TUNEL staining (arrows) which labels apoptotic nuclei. The sections were co-labeled with various cellular markers (red) and 6E10 antibody and counter-stained with DAPI. No apoptotic oligodendrocyte (C3) or motor neurons were found (C4), only a few microglia (C1) and astrocytes (C2) were positive for TUNEL. Insets of C1-3 show higher magnification of TUNEL positive nuclei in various cell types. C5 shows many TUNEL positive nuclei in dorsal horn at 7 days after spinal cord contusion injury as a positive control. MNs: motor neurons. Scale bar in A = 200μm, C = 100μm.

We immuno-labeled spinal cross-sections from 27 week old 5xFAD mice and age matched wild type (WT) littermates with anti-ChAT antibody (Fig 2A 1&2) to identify motor neurons. We counted the number of motor neurons in cervical cord, where the greatest number of plaque was found. Quantitative results revealed no difference between the total number of counted motor neurons in 5xFAD mice versus WT littermates (p = 0.08, Fig 2B). We further confirmed this finding using an apoptosis labeling kit (Fig 2C) which consistently showed at most only one or two TUNEL positive nuclei in each cervical cross-section. Co-labeling with cellular markers suggested that most of these apoptotic cells were either Iba-1-positive microglia or GFAP-positive astrocytes. No TUNEL-positive neurons or oligodendrocytes were found.

### Beta amyloid-positive threads in white and grey matter

**Fig 3:**
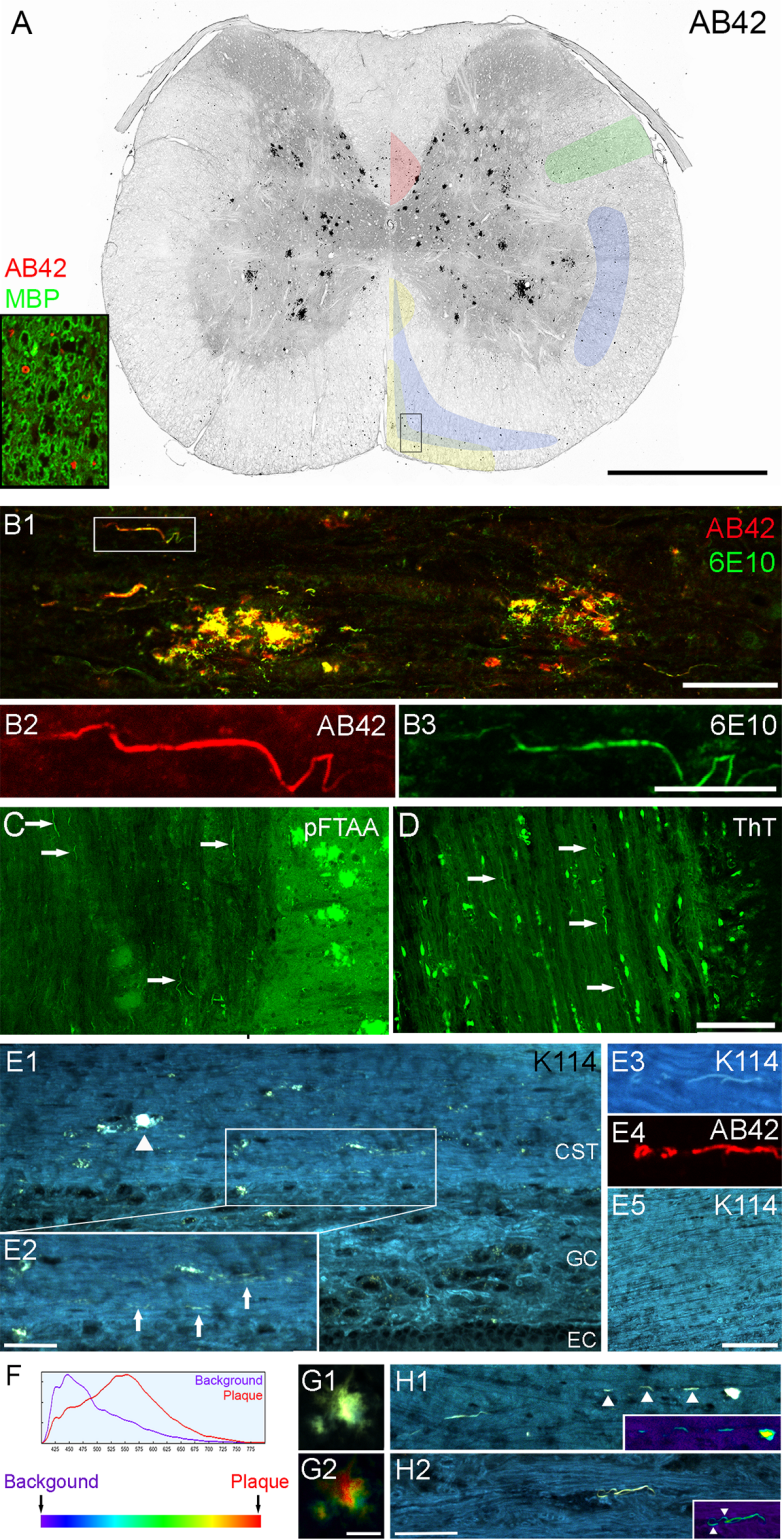
Beta amyloid-positive threads in 5xFAD mouse spinal cord. (A) Cervical spinal cross-section stained with AB42 (red) and myelin basic protein SMI99 (green) as shown in insert. For clarity of plaque and thread labeling only the AB42 staining is shown in the large cross-sectional image. The image was processed in grey scale and colour-inverted. Many black puncta were seen in the white matter, largely localized to the descending tracts outlined and colour-coded in the image as follows: corticospinal tract (red); rubrospinal tract (green); caudal and rostral reticulospinal tracts (blue); and medial and lateral vestibulospinal tracts (yellow). The outlines are based on the work of Watson and Harrison [19]. On closer examination, the puncta have unique structure such as thread or ring like as shown in insert (rectangle in cross-section). (B) Sagittal spinal section co-labeled with 6E10 (green) and AB42 (red) antibodies revealed thread structure and confirmed that they consisted of beta amyloid peptide (boxed area in B1 is enlarged in B2-3). (C-E) We used three conformationally-sensitive amyloid probes to confirm our findings. The threads are positive for pFTAA, ThT and K114 (arrows), indicating they possess beta sheet secondary structure. K114-positive threads (arrows in E2) are found in plaque laden (arrowhead) corticospinal tract (E1) and are positive for AB42 antibody (E3-4). No beta amyloid-positive threads are found in any wild type samples (E5). (F) K114 spectral emission red-shifts when bound to amyloid fibrils at high pH. (G) When truecolour images of K114-labeled plaques (G1) are converted to spectral pseudo-colour images (G2), it is clear that the emission spectrum of K114 varies considerably in different regions of a single plaque. (H) Truecolour images and the corresponding pseudo-colour (heat map) images (inserts) show spectral heterogeneity of K114 bound to amyloid threads as well. Arrowheads in H1 point to a broken thread; arrowheads in H2 point to the edge of the thread being more blue-shifted than the core. Scale bar in A = 1mm; B1, H1-2 = 50μm; B2-3 = 20μm; C-D, E5 = 100μm; E2 = 25μm; and G1-2 = 15μm. Abbreviations: background (BG); corticospinal tract (CST); ependymal cell layer (EC); grey commissure (GC).

Besides the obvious plaques, we observed a large number of punctate features in cross-sectional white matter in all plaque-laden samples stained with either 6E10 or beta amyloid 1–42 (AB42) isoform specific antibodies. Most had distinct features which excluded artifact such as antibody or tissue debris, and the puncta were primarily localized in descending tracts as shown in Fig 3A. To examine this further we processed additional spinal cord samples longitudinally. Since 6E10 can cross react with amyloid precursor protein (APP), we then co-labeled with both 6E10 and AB42. After formic acid or proteinase K treatment (an essential antigen retrieval step, S1 Fig), both antibodies reliably identified thread-like structures running in parallel with spinal axons (Fig 3B); these structures also survived proteolytic digestion with proteinase K at 10μg/ml for 30 minutes at 37°C (S1 Fig). Of note, thread-like structures were also found in the spinal grey matter, part of the ventral root close to the spinal cord, and in the dorsal root entry zone, but not in the dorsal root or its ganglia, peripheral nerve or muscle (S2 Fig). To examine whether these threads contain beta sheets, typical of conventional plaques [18], we used three conformationally-sensitive fluorescent amyloid probes (pFTAA, ThT and K114) [15–17]. In order to preserve any weak binding that may be informative, we either omitted the differentiation step involving lithium carbonate wash (K114) or only briefly rinsed the sections after staining (pFTAA and ThT). All three probes labeled beta amyloid plaques and threads (Fig 3C–3E) and the threads labeled by K114 were confirmed to match AB42 immuno-labeling (Fig 3E 3&4). Importantly, neither plaque-specific fluorescence nor thread-like structures were found in WT animals examined up to 37 weeks of age (Fig 3E5).

### K114 spectral signature of beta amyloid plaques

Interestingly, we found that K114 exhibited varying emission spectra even within a single plaque at pH 10.5 (S3 Fig) but not at acidic or neutral pH (data not shown). We therefore imaged a number of plaques and identified the most red-shifted spectrum at pH 10.5, which was used as the most pathological “plaque” reference spectrum, along with “background” spectrum from the K114 stained background in WT mice spinal cord to perform a quantitative spectral analysis (see Methods). We pseudo-coloured the range from violet to red to represent the increasingly red shifted, amyloid-specific spectra (Fig 3F) and analyzed our plaques and threads using this spectral processing scheme. Imaging with 405 nm excitation showed that beta amyloid plaques exhibited significant regional heterogeneity even within a single plaque (Fig 3G). Spectra for beta amyloid threads fell mostly in the middle of the range but heterogeneity was also observed, with the edges showing a more blue-shifted fluorescence (Fig 3H).

### Location of beta amyloid threads

**Fig 4:**
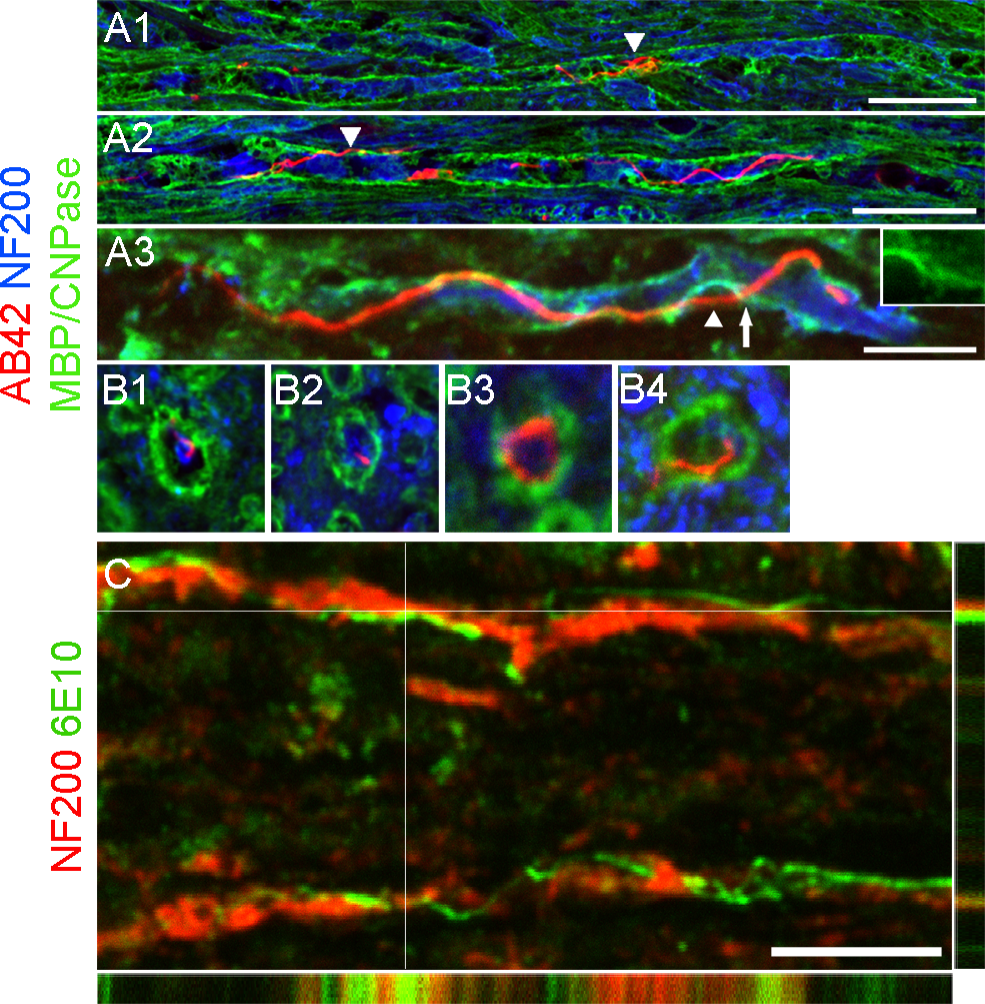
Most threads are located in the peri-axonal space. (A) Micrographs showing high magnification of sagittal spinal sections co-labeled with AB42 (red), neurofilament (blue) and myelin basic protein (A1-2; green) or CNPase (A3; green). The majority of threads are confined within the myelin cylinder. A thread coiled into a knot-like structure is visible in A1. A2 shows a long and slender thread running in parallel with an axon. Occasionally, threads are found outside myelin (arrowheads in A2 and 3). A3 shows part of the thread presumably inside the axon and part of it outside (arrowhead) and it appears to pass through an opening in the myelin (arrow and insert). (B) Cross-sectional images depicting threads outside (B1), inside (B2), and surrounding (B3) axons, as well as piercing through the myelin sheath (B4). (C) z-stack image depicting a thread (green, intersected lines) lying outside an axon (red). Scale bar in A1-A2 = 25μm; A3 = 10μm; B1-3 = 10μm in length; C = 10μm.

To more precisely identify the location of these threads in the white matter tracts, we immuno-labeled sagittal sections with myelin basic protein to visualize the myelin sheath, neurofilament for the axon compartment and AB42 for threads (Fig 4A). Threads were largely confined within the myelin cylinder, and had a slender and undulating configuration. They also appeared as coils or loops within the myelin. We were also able to find threads partly within the axonal compartment and at times appearing to pierce through the myelin sheath (Fig 4A3). Cross sectional staining revealed similar results (Fig 4B). At higher magnification, threads were seen lying adjacent to axon cylinders with minimal overlap (Fig 4C). Taken together, we believe that most of the threads are localized in the peri-axonal space, with some penetrating the myelin sheath, occasionally emerging into the extracellular space.

### Beta amyloid threads appear before plaques

**Fig 5:**
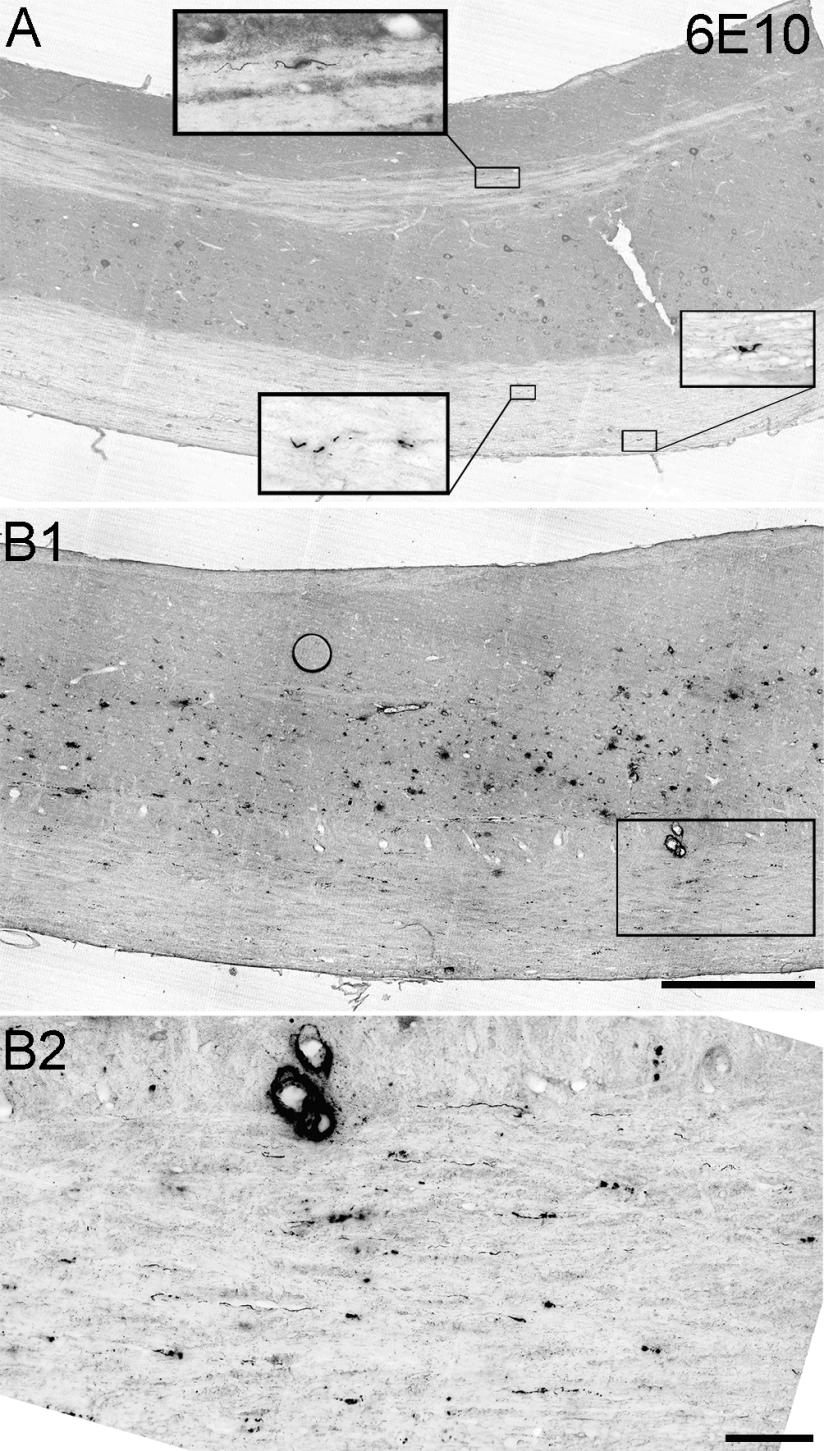
Threads appear before plaque deposition. (A) No plaques were found in 8 week old 5xFAD mouse spinal cord, however, 6E10-positive threads can be found (boxed areas) in this sagittal cervical cord section. Only 3 threads were found over the entire length of the cervical cord section. (B) In contrast, at 19 weeks of age numerous plaques were observed in the grey matter at the cervical level in 5xFAD mouse spinal cord (B1), and many threads were observed in the white matter (boxed area in B1 enlarged in B2). Scale bar in A and B1 = 500μm; B2 = 100μm.

Because of the more immature spectral signature of threads compared to plaques (Fig 3), we hypothesized that the former appear first, before deposition of larger plaques. We therefore examined younger 5xFAD mice. As mentioned earlier, plaques were first found in 11 week old mouse cervical cord. In contrast, we found threads as early as 8 weeks in the white matter at this spinal level prior to the appearance of plaques (Fig 5A), supporting the notion that threads indeed precede plaque formation. Moreover, increasing numbers of threads can be found in the spinal cord as plaque numbers increase (Fig 5B).

### Interaction with glial cells

**Fig 6:**
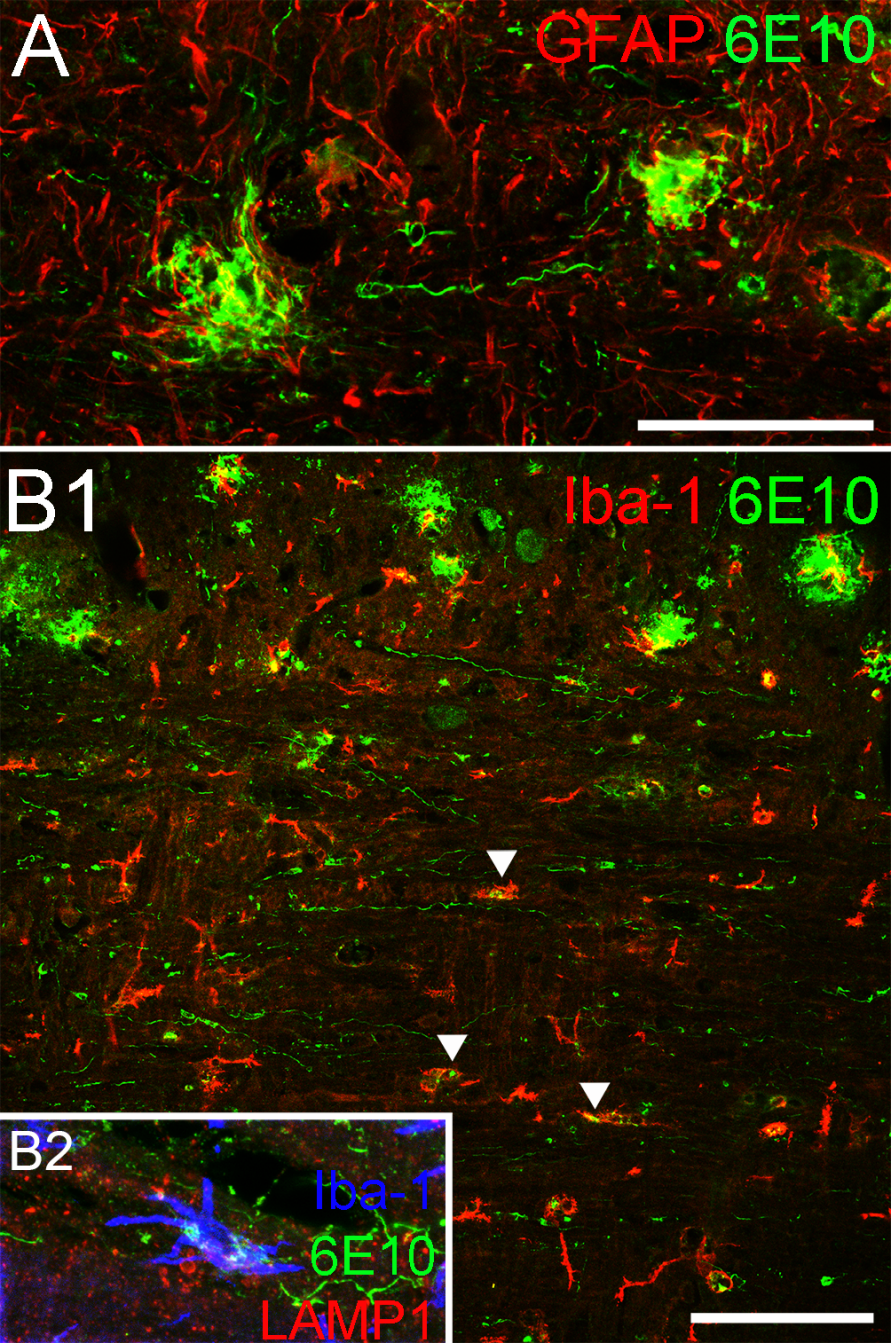
Interactions between threads and glial cells. (A) GFAP-positive astrocytes (red) are found surrounding two 6E10-positive plaques in this micrograph, but the astrocytes do not show a similar association with 6E10-positive threads. (B) Similarly, Iba-1-positive microglia are typically found in proximity or contact with plaques in the grey matter but do not appear to interact with threads to the same extent, although 6E10-positive deposits can be found inside some microglia in the white matter (arrowheads). Co-labeling with endosome antibody LAMP-1 (red), Iba-1 (blue) and 6E10 (green) reveals these deposits confined within endosomes inside microglia, which indicates that microglia are taking up amyloid deposits in the white matter tract. Scale bar in A = 50μm; B1 = 100μm.

Similar to the brain [9], beta amyloid plaques are highly engaged with astrocytes and microglia (Fig 6). We asked whether the threads might also be recognized by these glial cells. Immunostaining showed that astrocytes (Fig 6A) and microglia (Fig 6B1) in the white matter did not seem to preferentially associate with threads, although 6E10 immuno-reactivity was often seen inside microglia. Co-labeling with lysosome marker LAMP1 revealed overlap with 6E10-positive structures, suggesting that some amyloid deposits may be taken up by microglia and deposited in endosomes (Fig 6B2). However we could not determine the source of these amyloid inclusions (threads, plaques, or both).

### Threads are found in axonal spheroids in aged 5xFAD mice

**Fig 7:**
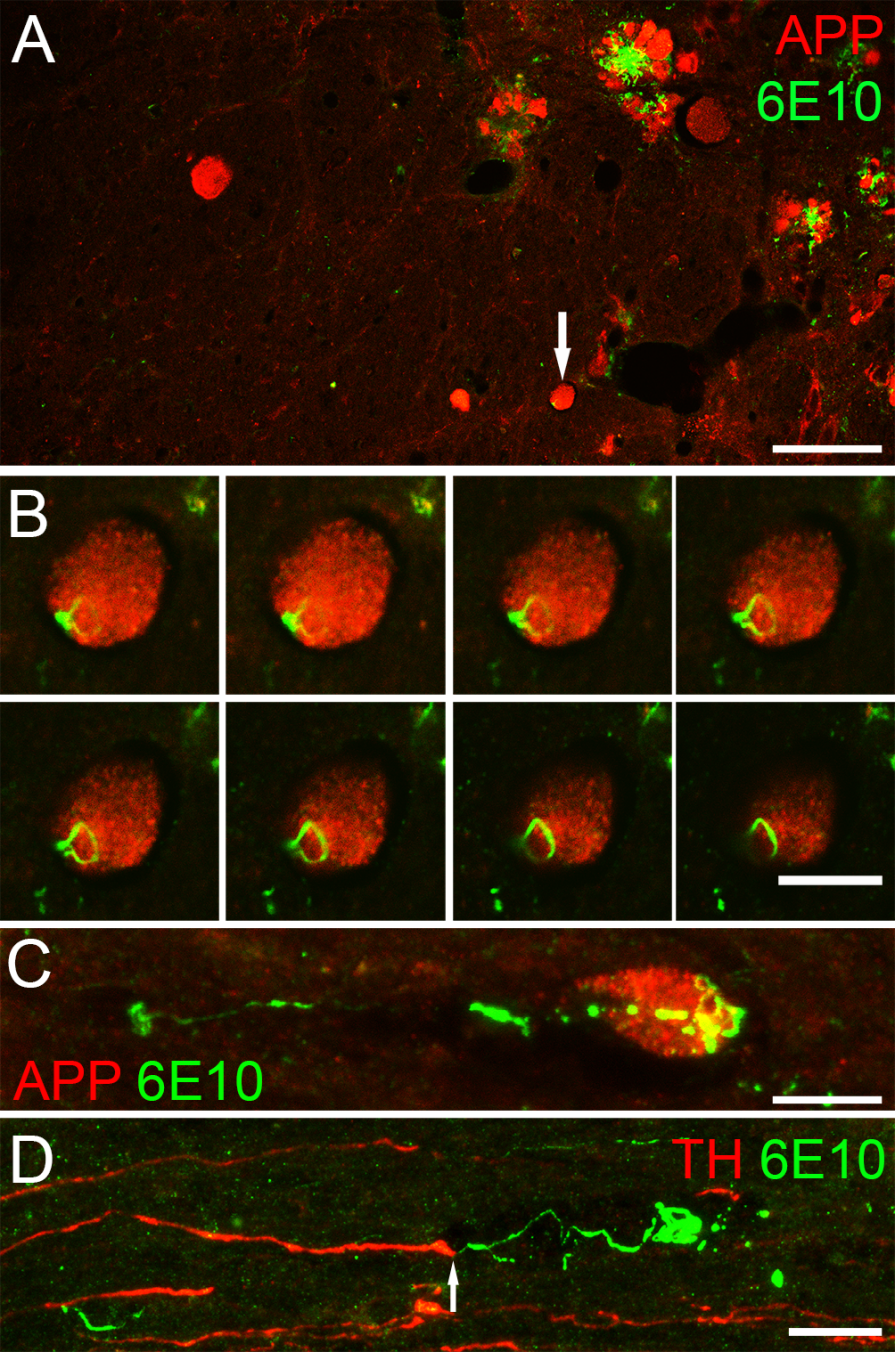
Threads may cause damage to axons. (A) Many APP-positive axonal spheroids (red) can be seen in a representative cross-section from a 27 week old 5xFAD mouse. 6E10-positive threads (green) are often seen co-labeling with APP staining (arrow) at this age. (B) Z-stack images (0. 5μm optical sections) from the spheroid indicated by the arrow in A reveal that a thread lies on the outside of the spheroid rather than inside it. (C) Similarly, a sagittal section showed a 6E10-labeled (green) thread at the tip of an APP-positive (red) spheroid. (D) Sagittal section stained with the aminergic fibre marker, tyrosine hydroxylase (red), and 6E10 (green) shows a thread, extending from a spheroid at the end of an aminergic fibre, that forms a coil about 30μm caudal to the spheroid. Scale bar in A = 50μm; B-C = 10μm and D = 15μm.

APP accumulates in axonal spheroids in injured axons [20]. In our tissue, increasing numbers of axonal spheroids were seen in aged 5xFAD mice (Fig 7A). We noticed that beta amyloid threads were present in approximately half of these axonal swellings in cross-sections. Optical sectioning revealed that threads were mainly located near the surface of spheroids (Fig 7B) and when viewed longitudinally, threads could occasionally be seen forming coils at the tip of the swelling (Fig 7C). Threads were also occasionally observed running in parallel with tyrosine hydroxylase (TH) positive aminergic axons (Fig 7D). Since aminergic fibres are most likely of supraspinal origin, we were certain that it is a descending axon. In one example (Fig 7D), a thread appeared to extend from the tip of a TH-positive axonal spheroid (arrow) and form a coil about 30 μm caudal to the spheroid. It is possible that the TH positive axon retracted and the thread was coiling to form a bigger deposition.

### Age-dependent myelin structural change in 5xFAD mice

**Fig 8:**
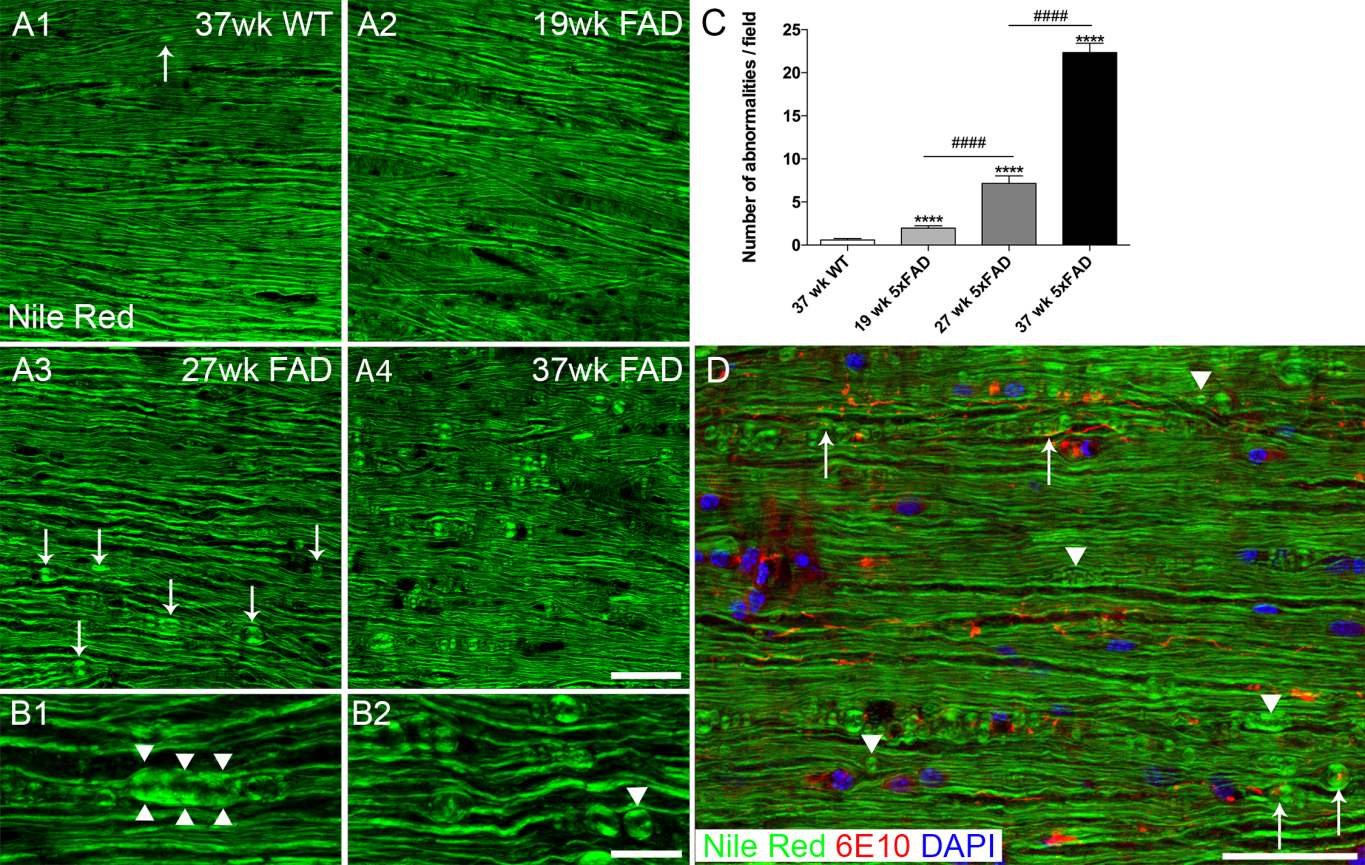
Myelinopathy in 5xFAD mouse white matter. Representative images showing Nile Red labeled spinal cord ventral white matter. 37 week old wild type mouse (A1) sample shows normal appearing myelin staining profile. Very infrequent myelin spheroid (arrow) can be seen in old wild type samples. Myelin staining appears normal in 19 week old 5xFAD mouse spinal cord (A2), but abnormalities are clearly visible at 27 weeks (A3) and are more prevalent in 37 week old 5xFAD mouse samples (A4). For example, myelin spheroid/ thickening (B1 arrowheads) and sphere formation (B2, arrowhead) are observed in a 27 week old 5xFAD mouse sample. Quantitative analysis of myelin abnormalities at 19, 27, and 37 week old 5xFAD mice and 37 week old WT mice (C). Results showed a progressive and significant increase of myelin abnormalities from 19 week to 37 week old 5xFAD. ****, p<0.001, ####, p<0.001. (D) Merging of the Nile Red (green) image with 6E10 (red) and DAPI (blue) images reveals that myelin anomalies are found in contact or close proximity to threads (arrows), but also in the absence of threads (arrowheads). Scale bar in A = 50μm, B = 20μm and D = 100μm.

Besides the interaction of beta amyloid threads with axons, we asked whether they also affect myelin. We used the lipophilic probe Nile Red to label myelin since this dye yielded a more homogeneous staining of the myelin sheath compared to immunohistochemistry. All WT samples (examined up to 37 weeks of age) showed uninterrupted, normal appearing myelin staining profiles with myelin swelling seen only on rare occasions (Fig 8A1). In 5xFAD mice, myelin appeared normal in 19 week old or younger samples (Fig 8A2). By 27 weeks however, abnormalities in myelin were readily observed (Fig 8A3), and included myelin spheroids (Fig 8B1) and individual spheres (Fig 8B2 arrowhead). In 37 week old 5xFAD mice, myelin abnormalities were even more prevalent (Fig 8A4). Quantitative analysis showed progressive and significant increase of myelin abnormalities as animal age (Fig 8C). Although myelin swellings were occasionally found in proximity to beta amyloid threads, this was not a consistent finding, so there was no evidence of a spatial relationship between threads and myelin swellings overall (Fig 8D).

## Discussion

Beta amyloid plaques can be found in human spinal cord, most prominently in familial AD cases [2,3]. However, no studies thus far have provided a detailed description of beta amyloid plaque load in the spinal cord. As an integral part of the CNS, the spinal cord possesses the same molecular machinery for forming beta amyloid plaques as demonstrated in various transgenic mouse models over-expressing either APP mutation alone or both APP and PS1 mutations [11,12,21]. In this study, we characterize spinal cord pathology in one of the commonly used transgenic mouse models, the 5xFAD mouse line, which expresses mutated APP and presenilin 1 driven by the neuronal Thy1 promoter [9]. 5xFAD mice show plaque deposition as early as 2 months in the brain with behavourial deficits becoming apparent at around 5 months of age; therefore the rapid and massive accumulation of Alzheimer’s pathology makes this mouse line an attractive and popular model [9]. Our study focused on the cord revealed findings that share some similarities with the brain. First, most of the plaques deposit in the grey matter but some are found in the white matter and especially the corticospinal tract. Of note, deposits in the white matter have also been reported in human AD brain [22]. Second, as in brain, there is a time dependent increase in plaque load in the cord; third, and perhaps most interestingly, beta amyloid plaques are not distributed homogeneously along the length of the spinal cord, with more plaques in cervical cord at all time points examined. Our study also revealed long and thin beta amyloid positive threads running in parallel with the axons in the white matter, and provided evidence that these threads might contribute to axonopathy in the spinal cord.

Beta amyloid plaque deposition first appeared in the two enlargements in spinal cord (cervical and lumbar) with notably more plaques in the former. This was unexpected because spinal levels are repetitive and structurally similar, especially comparing cervical and lumbar levels. The non-uniform distribution of plaques may be due to a number of factors. First, it has been shown that increasing synaptic activity enhances beta amyloid secretion [23], therefore amyloid deposition might be activity-dependent. With more motor neurons located in the two enlargements, particularly in the cervical segments for control of fine movement of the forelimbs and digits, this could account for the earlier and more pronounced plaque deposition in this region. A second possible explanation is suggested by the absence of plaques in Rexed laminae I and II in our and other studies [24], neurons in these two layers relay sensory information to the brain, suggesting that descending supraspinal innervation plays a role in plaque formation. Given the importance of these two enlargements for motor control, it is more likely that they receive more descending inputs and therefore accumulate more plaques; this possibility is in line with Yuan’s study where ablation of the unilateral sensorimotor cortex in TgCRND8 mice overexpressing human APP, resulted in a reduction of amyloid plaques in the contralateral side of the spinal cord [12], indicating that one of the sources of beta amyloid is likely from the cortex itself. Despite the fact that lower motor neurons also express the transgenes, more plaques can be found in the intermediate grey than in the motor neuron clusters suggest motor neurons did not play a major role in local plaque synthesis. Together with the evidence of plaques in the CST, it is likely that the cortical source contributed to (either directly by transport of precursors or indirectly by descending electrical activity) a considerable portion of plaque formation in the spinal cord. Lastly, these two enlargements contain central pattern generators that are able to produce synchronous activity for coordinated rhythmic movement. It has been suggested that the associative connectivity of neurons and the overall pattern of activity, rather than merely the intensity of activity, drives plaque deposition [25]. This may also explain why laminae I and II are essentially plaque free, because the major function of neurons in these two layers is to relay sensory inputs to the brain or deeper parts of the spinal cord, so rhythmic activity is limited in those structures.

Beta amyloid protein is the proteolytic product of APP, which is anterogradely transported to the nerve terminals where amyloid plaques are more likely to be formed to regulate synaptic activity [23,26,27]. Beta amyloid can also form in axonal compartments as demonstrated in an ex vivo model [28]. Indeed the presence of plaques along the corticospinal tract, which consists of descending axons originating at the motor cortex, strongly supports this notion [12]. Using spinal cord sagittal sections, we identified long (>100μm) beta amyloid positive thread-like structures, that are beta sheet rich, and resistant to enzymatic degradation (S1 Fig). They are possibly derived from APP which is aberrantly cleaved into beta amyloid and secreted outside the axon through a recently discovered misfolding-associated protein secretion pathway due to compromised proteosomal degradation machinery [29]. The fact that they are lying juxtaposed to axons inside the myelin sheath suggests that the physical constraint imposed by the still-intact myelin structure may limit the ability of amyloid deposits to form plaques within the white matter. The myelin sheath might also protect the threads from detection and subsequent removal by microglia [2], thereby facilitating their growth over time. It is reasonable to speculate that as a thread grows in size, it may coil and occupy more space, eventually mechanically transecting the axon within the myelin sheath and producing an axonal spheroid at the transected end (Fig 7C & 7D). The colocalization of beta amyloid threads with spheroids may indicate that spheroids contribute to the deposition and/or growth of the threads by providing accumulating APP that acts as a continuous source of beta amyloid. However, given that the threads are present long before any spheroids can be observed, it is more likely that the threads damage the associated axons and induce spheroid formation, which may then contribute to further thread growth. We speculate that once an axon degenerates and demyelinates, it could leave a protease-resistant beta amyloid seed that can eventually grow into a more traditional plaque in white matter tracts (Fig 1A).

The majority of threads were found in white matter tracts while some were found in the grey matter. Only a few threads were found in ventral roots in close proximity to the ventral root exit zone but none were found in peripheral nerve such as the sciatic nerve (S2 Fig). This is unexpected because spinal motor neurons also express the mutated proteins and intracellular beta amyloid was seen in immuno-labeled samples. Similarly, we did not find any threads in the dorsal root but threads were found in the dorsal root entry zone where incoming fibres first encounter CNS-resident astrocytes. It is possible that Schwann cells, the myelinating cells of the peripheral nervous system, remove debris more efficiently than CNS oligodendrocytes [30]. The phagocytic ability of Schwann cells is well documented and given their intimate location vis-à-vis myelin, it is possible that these glia remove the very early, still protease-sensitive amyloid deposits before aggregation proceeds to the more mature axon-damaging and plaque-forming threads as seen in the CNS. This may also explain why no amyloid plaques are found in peripheral nerves, or at the neuromuscular junctions (S2 Fig). We examined 5XFAD brain sections and found threads were also present in the grey and white matter of the brain (S1 and S4 Figs). However, since the axons in the more prominent tracts such as corpus callosum and external capsule are of smaller calibre, thread-like amyloid pathology is more difficult to detect in these regions. Similar descriptions of thread-like structures have been reported previously. For example, using Gallyas silver stain, Braak and Braak described slender threads in pyramidal cells [31] and Ashford et al. found threads that “appeared to have broken out of the dendrite” using an antibody against paired helical filament [32]. Although similar to the thread-like structures reported here, those earlier reports described threads found in the neuropil only. A more related finding from Christensen *et al*. described beta amyloid structures at close proximity to axonal spheroids in the APP/PS1 knock-in mouse [6], which strongly resembles our findings in aged 5xFAD mice, suggesting that this phenomenon is not restricted to a single Alzheimer’s mouse model. Regardless of constituents, noteworthy is the ability of these thread-like structures to pierce through membranes, such as dendrites or myelin, thus potentially causing damage to these structures, perhaps by purely mechanical means.

In addition to immunohistochemistry, we used three conformationally sensitive amyloid probes to investigate the protein structure of these threads. The reliance of beta sheet structure for these probes to fluoresce was demonstrated by the treatment with formic acid which abolished amyloid plaque and thread staining (S5 Fig). Amongst them, K114 was most informative because of its ability to emit different spectra in each and within plaques. K114 is a Congo red analogue and exhibits an increase in fluorescence intensity when bound to amyloid fibrils at neutral pH [17,33] and spectral shifts in both excitation and emission upon binding to amyloid at high pH (>pH 9.5) [33]. At pH 10.5, K114 tends to form a highly fluorescent phenolate species with fibrils, and for this reason we used an alkaline pH in our experiments. Using K114, we observed heterogeneity between plaques and even within individual plaques, with such spectral shifts likely reflecting different configurations of beta amyloid assembly. In support of this, Condello et al. identified plaque micro-regions labeled by AB42 antibody and protofibrillar beta amyloid binding substance Curcumin while the core of the plaque was more homogenously labeled by anti-amyloid 1–40 antibody [34]. Since these probes can label other amyloid proteins such as misfolded tau, we used two phosopho-tau antibodies, namely AT8 and phospho-tau at Serine 262, but we failed to detect any hyperphosphorylated tau in our 5xFAD samples (S6 Fig). We used the P301L tau mutant as a positive control [35] and confirmed AT8 and phosphor-tau (Ser262, not shown) positive staining in cortical regions. This The lack of tau staining in 5xFAD mouse is consistent with other findings, as rodent AD models generally fail to replicate the prominent tau pathology seen in humans with AD. Taken together, our results indicate that these threads are beta sheet rich and possess a spectral signature that closely resembles those of amyloid plaque, which suggests that these threads may act as the origins of plaques, at least in the white matter of the CNS.

Myelinopathy in AD is becoming more recognized. For example, the myelin staining profile in CA1 hippocampal region and superficial layer II/III of the entorhinal cortex in 3xTg-AD mice is compromised compared to age matched control, indicating a regional disruption of myelin in this mouse model of AD, with ultrastructural studies also revealing granulation of myelin [35]. Similarly, magnetic resonance imaging in humans reveals a reduction in white matter in early and established AD, implying that myelinopathy might also be an important pathophysiological component in clinical AD [36–38]. However, a clear image of morphological change in myelin associated with AD has generally been lacking even in mouse models. To acquire high definition images of myelin sheath, we used the lipophilic dye Nile Red. We observed substantial myelinopathy in the spinal cord white matter in aged 5xFAD mice with very little myelin pathology in age-matched WT littermates. Morphological abnormalities include myelin spheroids and myelin micelle formation and it is evident that these lesions increased over time. It is not known whether the threads directly cause damage to myelin, or whether axonal pathology secondarily causes myelin breakdown.

Motor deficits have been reported but only in older transgenic AD mice including the 5xFAD and TgCRND8 strains [12,13]. This contrasts with amyloid pathology including thread-like deposits in spinal white matter at much younger ages in the 5XFAD mouse. This discrepancy can be readily explained by the very non-linear relationship between number of remaining functional axons in the cord vs sensori-motor disability that emerges only when the majority of spinal axons are injured or lost. Thus, although amyloid pathology is detectable early in the 5XFAD cord, there is sufficient reserve in spinal tracts so that clinical deficits do not manifest until later.

Our study provides a spatio-temporal map of amyloid plaque load in the 5xFAD mouse spinal cord, and suggests an underlying mechanism of amyloid deposition that may lead to plaque formation, particularly in the CNS white matter. The novel beta amyloid-containing thread-like structures identified in this study were intimately associated with spinal axons and may act as an initial source of amyloid burden and secondary axonal damage in CNS white matter. It is worth mentioning that the 5xFAD mouse model is an aggressive transgenic AD model therefore cord pathology as described here may be more prominent compared to less aggressive transgenic lines. On the other hand, human AD progresses over decades allowing ample opportunity for development of analogous pathology. Further studies using human AD tissue will be required to confirm similar pathology in the human. Collectively, we showed axonopathy and myelinopathy in 5xFAD mouse that might impede signal propagation and affect normal function, underscoring the importance of primary white matter pathology in Alzheimer’s and related diseases.

## Supporting information

S1 Fig Formic acid or proteinase K treatment is required to reveal threads in frozen sections. No thread structure can be seen in the white matter tract without formic acid treatment is performed (A1). Treating samples with 98% formic acid for 5 minutes at room temperature before performing immunohistochemistry unmasked the beta amyloid thread antigenic sites (A2). We also tested whether the threads are resistant to proteinase degradation. We incubated our formic acid treated section in 10μg/ml of proteinase K in Tris-EDTA- calcium chloride buffer (pH 8.0) at 37°C for 30 minutes before performing immunohistochemistry. Thread structures can still be seen after treatment suggesting that threads are resistant to proteinase degradation (A3). In fact, proteinase K treatment alone is also sufficient to unmask threads staining (B & C). Incubation of adjacent brain sections from 27 week old 5xFAD mouse in aforementioned buffer at 37°C for 30 minutes did not reveal threads (B1-3, B2 and B3 are higher magnification images in boxed region in B1 and B2) whereas treatment with 10μg/ml proteinase K, without formic acid treatment, revealed extensive thread staining in substantia nigra pars reticulata (C1-3). Scale bar in A = 100 μm; B1 & C1 = 1 mm; B3 & C3 = 100 μm.
(TIF)

S2 Fig No threads are found in peripheral tissue. Apart from the CNS, we also surveyed the peripheral nervous system and muscle with 6E10 antibody in 5xFAD mice. No threads were found in dorsal root (DR), dorsal root ganglion (DRG), sciatic nerve (SN) or at neuromuscular junctions in muscles (Mus). Only a few short threads were found in ventral root (VR) near the spinal cord exit zone. However, we could find 6E10-positive (green) threads (arrows) in the dorsal root entry zone (DREZ) where the ascending fibres enter into the CNS, GFAP staining (red) revealed where the astrocytes stop at the DREZ. Scale bar in A- F = 100μm.
(TIF)

S3 Fig Variability in K114 spectral emission within and among plaques in the spinal cord. (A) We determined the background spectra and the most red-shifted spectra from our K114 stained spinal cord (as described previously). We then pseudo-coloured the plaques using those reference spectra, such that red indicates greater pathology and blue/violet indicates less pathology. (B) An image showing a cervical spinal cross-section from a 27 week old 5xFAD mouse. We randomly selected plaques (boxes a-h) for higher magnification scanning. Using a spectral detector collecting at 430-750nm, we found spectral heterogeneity among the plaques and even within individual plaques. True colour images for individual spinal cord plaques are depicted in a-h, whereas the corresponding pseudo-coloured images are depicted in a’-h’, respectively. A similar staining pattern and spectral signature can be found in plaques in the brain (i, j), although pseudo-colour images of the latter (i’, j’) tend to have a larger area with a more uniformly red-shifted K114 spectrum. We speculate that it is due to the difference in composition of beta amyloid species in the brain compared to the spinal cord. Scale bar a-h = 50μm, i = 100μm and j = 50μm.
(TIF)

S4 Fig Threads are also found in 5xFAD mouse brain. (A) 6E10-labeled cortical 5xFAD sample is filled with amyloid plaque. Plaques are over-exposed to reveal the threads which are weaker in intensity. They resemble the threads in the spinal cord in terms of morphology, length and thickness. (B) Threads in the external capsule are also positive for K114. The boxed area is a maximum intensity projection from a z-stack image. Scale bar in A = 100μm and B = 50μm.
(TIF)

S5 Fig Pre-treatment with formic acid abolished ThT and pFTAA staining in amyloid plaques and threads. 11 week old 5xFAD coronal brain (A) and sagittal spinal cord cervical (B) adjacent sections were treated with or without formic acid for 5 minutes before staining with 0.5% ThT. No plaque staining was seen after formic acid treatment. Similarly, 27 week old 5xFAD mouse sagittal cervical spinal cord sections were incubated with or without formic acid before staining with 3μM pFTAA (C). Staining in plaques and threads (inset in C1, colour inverted and converted to black and white for easy visualization) was only been without formic acid treatment. Scale bar for A = 1 mm, B = 500 μm, and C = 200 μm.
(TIF)

S6 Fig Beta amyloid thread is not tau positive. 11 week old 5xFAD mouse spinal cord section was stained with AB42 (A1) and phospho-tau antibody AT8 (A2). No AT8 staining is found in any regions. To verify AT8 antibody specificity, we used a transgenic model expressing human P301L mutant tau. Results showed that phospho-tau positive neurons can be found in 14 month old mouse cortical layers (B2). No AB42 positive staining was found in the same section (B1). Scale bar in A = 100 μm, B = 150 μm.
(TIF)
