# Supplementary figures and images for: Axonal and myelinic pathology in 5xFAD Alzheimer’s mouse spinal cord
Source: PLoS One. 2017 Nov 27;12(11):e0188218. doi: 10.1371/journal.pone.0188218 (PMC5703477; doi:10.1371/journal.pone.0188218)

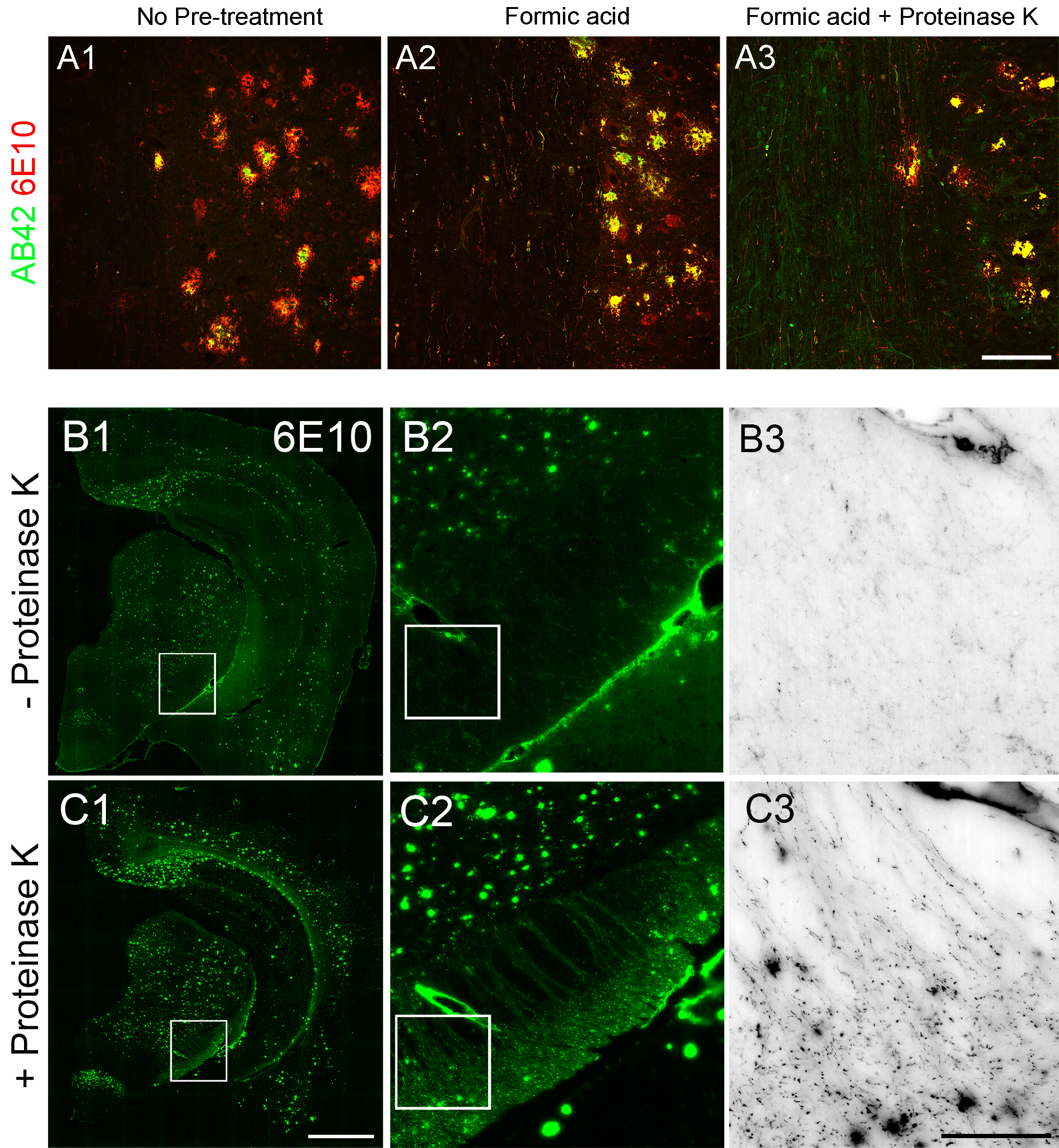

Supplement: S1 Fig — No thread structure can be seen in the white matter tract without formic acid treatment is performed (A1). Treating samples with 98% formic acid for 5 minutes at room temperature before performing immunohistochemistry unmasked the beta amyloid thread antigenic sites (A2). We also tested whether the threads are resistant to proteinase degradation. We incubated our formic acid treated section in 10μg/ml of proteinase K in Tris-EDTA- calcium chloride buffer (pH 8.0) at 37°C for 30 minutes before performing immunohistochemistry. Thread structures can still be seen after treatment suggesting that threads are resistant to proteinase degradation (A3). In fact, proteinase K treatment alone is also sufficient to unmask threads staining (B & C). Incubation of adjacent brain sections from 27 week old 5xFAD mouse in aforementioned buffer at 37°C for 30 minutes did not reveal threads (B1-3, B2 and B3 are higher magnification images in boxed region in B1 and B2) whereas treatment with 10μg/ml proteinase K, without formic acid treatment, revealed extensive thread staining in substantia nigra pars reticulata (C1-3). Scale bar in A = 100 μm; B1 & C1 = 1 mm; B3 & C3 = 100 μm. (TIF) [file pone.0188218.s001.tif]

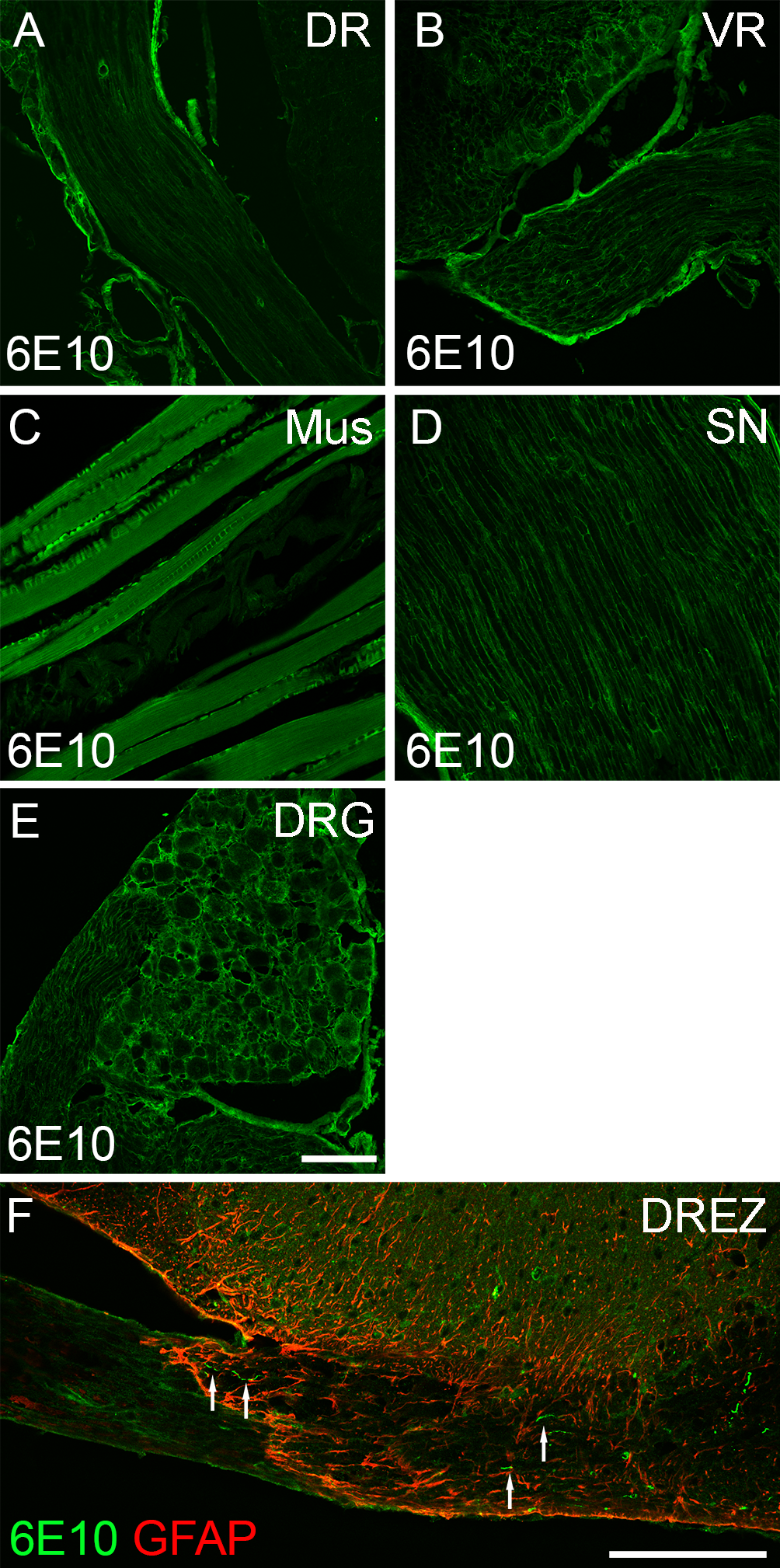

Supplement: S2 Fig — Apart from the CNS, we also surveyed the peripheral nervous system and muscle with 6E10 antibody in 5xFAD mice. No threads were found in dorsal root (DR), dorsal root ganglion (DRG), sciatic nerve (SN) or at neuromuscular junctions in muscles (Mus). Only a few short threads were found in ventral root (VR) near the spinal cord exit zone. However, we could find 6E10-positive (green) threads (arrows) in the dorsal root entry zone (DREZ) where the ascending fibres enter into the CNS, GFAP staining (red) revealed where the astrocytes stop at the DREZ. Scale bar in A- F = 100μm. (TIF) [file pone.0188218.s002.tif]

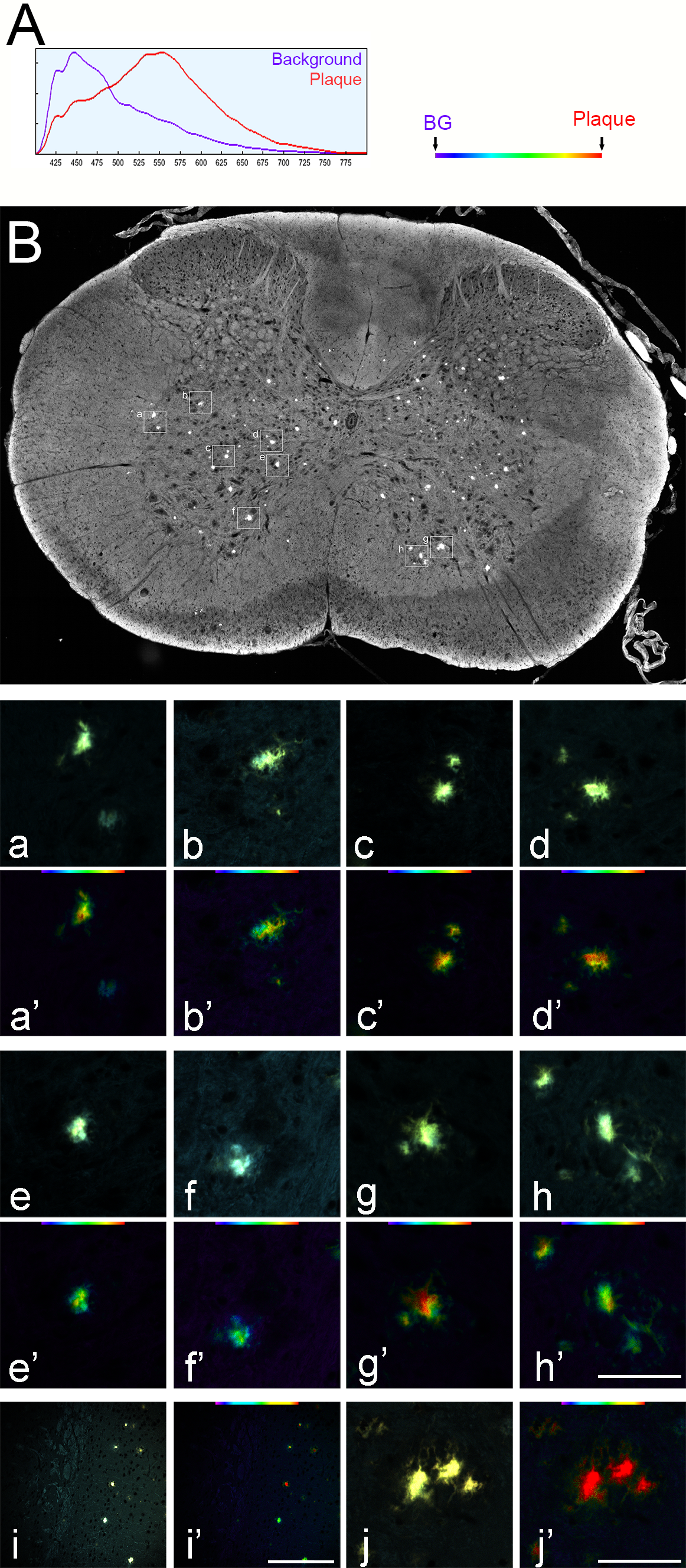

Supplement: S3 Fig — (A) We determined the background spectra and the most red-shifted spectra from our K114 stained spinal cord (as described previously). We then pseudo-coloured the plaques using those reference spectra, such that red indicates greater pathology and blue/violet indicates less pathology. (B) An image showing a cervical spinal cross-section from a 27 week old 5xFAD mouse. We randomly selected plaques (boxes a-h) for higher magnification scanning. Using a spectral detector collecting at 430-750nm, we found spectral heterogeneity among the plaques and even within individual plaques. True colour images for individual spinal cord plaques are depicted in a-h, whereas the corresponding pseudo-coloured images are depicted in a’-h’, respectively. A similar staining pattern and spectral signature can be found in plaques in the brain (i, j), although pseudo-colour images of the latter (i’, j’) tend to have a larger area with a more uniformly red-shifted K114 spectrum. We speculate that it is due to the difference in composition of beta amyloid species in the brain compared to the spinal cord. Scale bar a-h = 50μm, i = 100μm and j = 50μm. (TIF) [file pone.0188218.s003.tif]

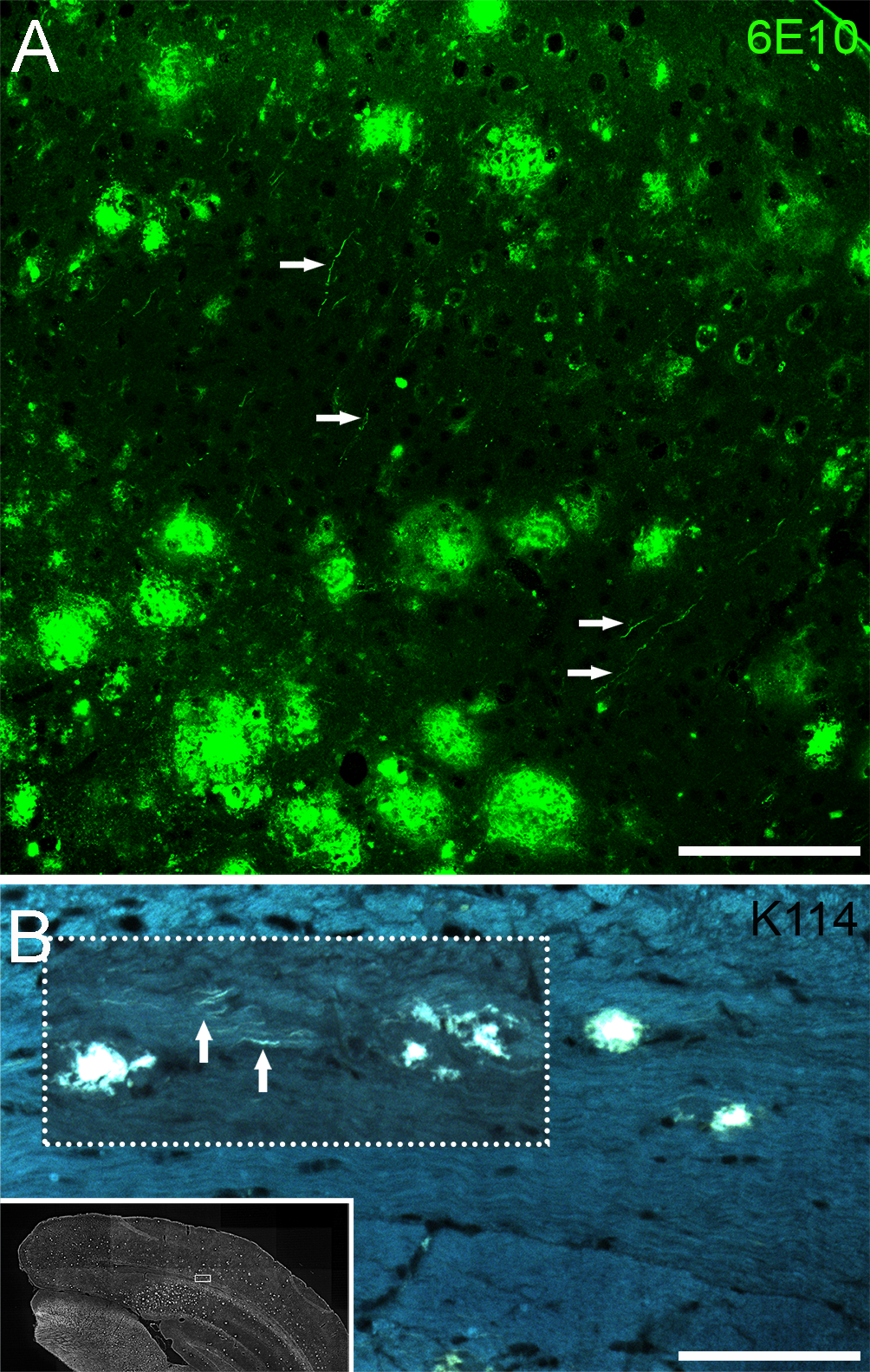

Supplement: S4 Fig — (A) 6E10-labeled cortical 5xFAD sample is filled with amyloid plaque. Plaques are over-exposed to reveal the threads which are weaker in intensity. They resemble the threads in the spinal cord in terms of morphology, length and thickness. (B) Threads in the external capsule are also positive for K114. The boxed area is a maximum intensity projection from a z-stack image. Scale bar in A = 100μm and B = 50μm. (TIF) [file pone.0188218.s004.tif]

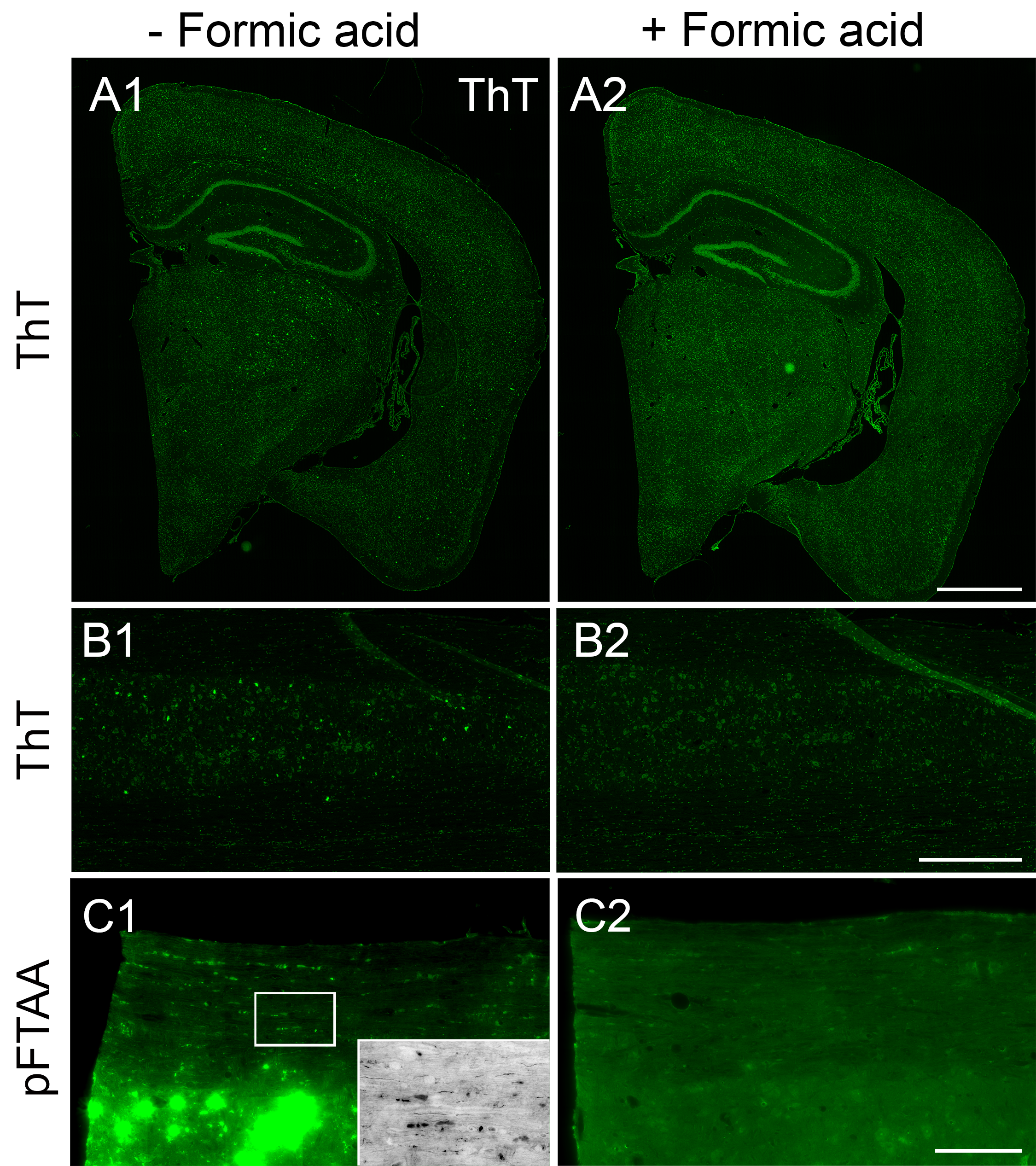

Supplement: S5 Fig — 11 week old 5xFAD coronal brain (A) and sagittal spinal cord cervical (B) adjacent sections were treated with or without formic acid for 5 minutes before staining with 0.5% ThT. No plaque staining was seen after formic acid treatment. Similarly, 27 week old 5xFAD mouse sagittal cervical spinal cord sections were incubated with or without formic acid before staining with 3μM pFTAA (C). Staining in plaques and threads (inset in C1, colour inverted and converted to black and white for easy visualization) was only been without formic acid treatment. Scale bar for A = 1 mm, B = 500 μm, and C = 200 μm. (TIF) [file pone.0188218.s005.tif]

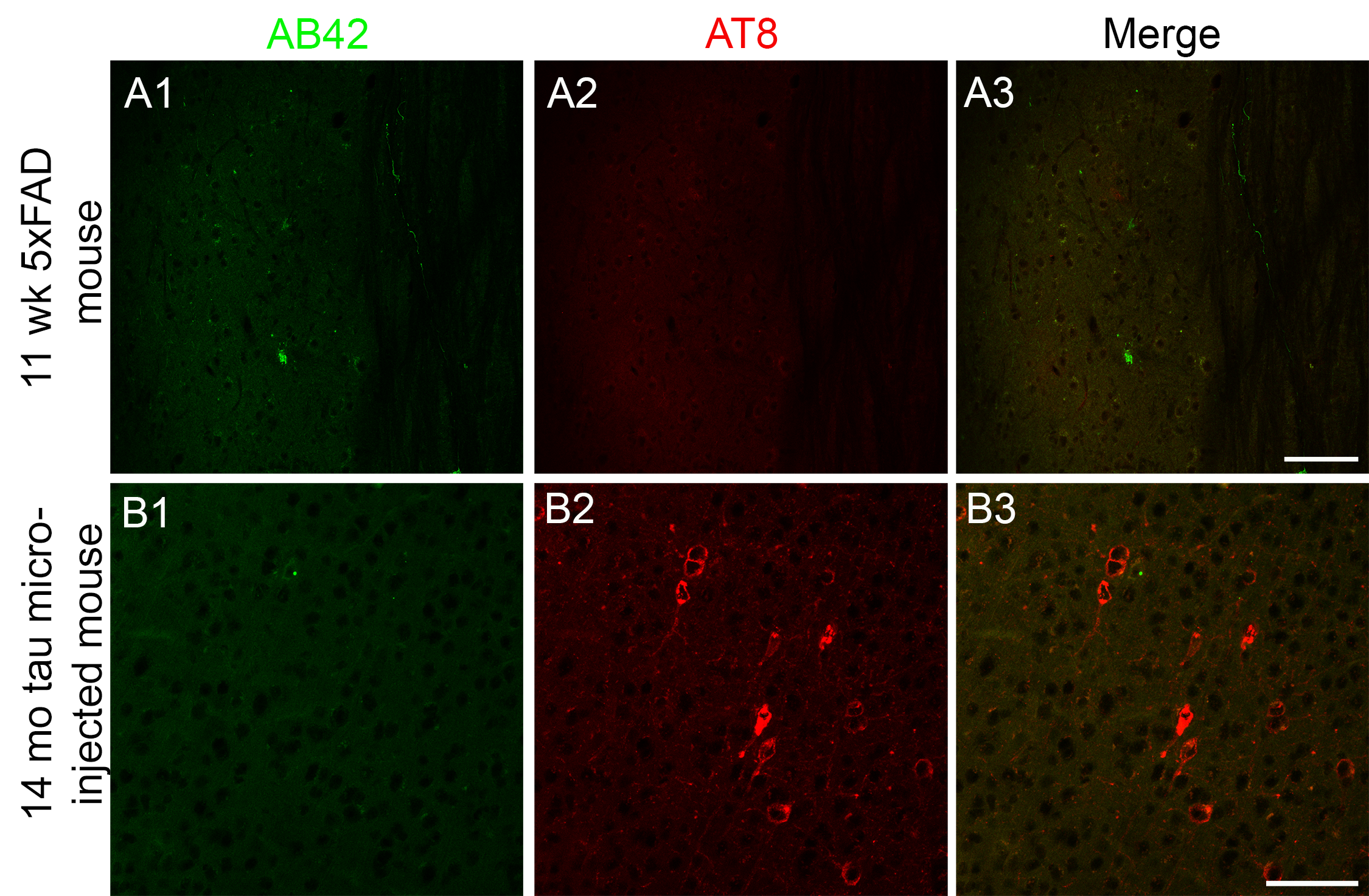

Supplement: S6 Fig — 11 week old 5xFAD mouse spinal cord section was stained with AB42 (A1) and phospho-tau antibody AT8 (A2). No AT8 staining is found in any regions. To verify AT8 antibody specificity, we used a transgenic model expressing human P301L mutant tau. Results showed that phospho-tau positive neurons can be found in 14 month old mouse cortical layers (B2). No AB42 positive staining was found in the same section (B1). Scale bar in A = 100 μm, B = 150 μm. (TIF) [file pone.0188218.s006.tif]
